# Seasonal effects on incidence and outcomes in idiopathic sudden sensorineural hearing loss

**DOI:** 10.3389/fneur.2026.1753066

**Published:** 2026-03-23

**Authors:** Jameel Ghantous, Shahar Barak, Tarnovsky Yehuda, Orr Israeli, Ayalon Hadar, Riki Salem, Jean-Yves Sichel, Ronen Perez, Chanan Shaul

**Affiliations:** Department of Otolaryngology and Head and Neck Surgery, Shaare Zedek Medical Center, Faculty of Medicine, Hebrew University of Jerusalem, Jerusalem, Israel

**Keywords:** corticosteroids, prognosis, seasonal variation, sensorineural hearing loss, sudden hearing loss

## Abstract

**Objective:**

To investigate whether seasonal onset of idiopathic sudden sensorineural hearing loss (ISSNHL) influences disease incidence and hearing recovery outcomes, addressing limited and conflicting evidence regarding seasonal prognostic factors.

**Methods:**

This retrospective cohort study analyzed 596 patients (mean age 51.5 ± 17.7 years, range 18–88 years) diagnosed with ISSNHL within 14 days of symptom onset at a tertiary referral center between 2012 and 2023. Patients were categorized by seasonal onset: spring (*n* = 145), summer (*n* = 140), autumn (*n* = 147), and winter (*n* = 164). All patients received standardized treatment according to AAO-HNS guidelines, including high-dose oral corticosteroids, with salvage intratympanic steroid injections for suboptimal responders. Audiometric outcomes included pure-tone average (PTA), speech recognition threshold (SRT), and word recognition score (WRS). A comprehensive literature review identified 14 relevant studies examining seasonal patterns in ISSNHL.

**Results:**

ISSNHL incidence was evenly distributed across seasons (spring: 24.3%; summer: 23.5%; autumn: 24.7%; winter: 27.5%; *p* = 0.534). No significant differences were observed in baseline demographics, comorbidities, or audiometric parameters across seasons (*p* > 0.7). Post-treatment improvements ranged from 8.62 to 10.48 dB for PTA, and 9.4 to 14.15% for WRS, with no statistically significant seasonal differences (*p* > 0.5). Monthly analysis revealed variation without consistent seasonal patterns. Multivariate analyses confirmed that season of onset was not an independent predictor of recovery.

**Conclusion:**

Seasonal onset does not significantly influence ISSNHL incidence or hearing recovery outcomes. This large-scale investigation, representing the most extensive single-center cohort examining seasonal effects on ISSNHL, challenges previous smaller studies suggesting spring-onset superiority and indicates that clinical management should focus on established prognostic factors rather than seasonal timing.

## Introduction

Idiophatic Sudden Sensorineural Hearing Loss (ISSNHL) is considered an otologic emergency and is defined as a rapid-onset hearing loss of at least 30 decibels (dB) across three contiguous audiometric frequencies, occurring within 72 h. Despite extensive research, the underlying etiology remains unclear in the majority of cases (1–3). The annual incidence of ISSNHL ranges from 5 to 20 cases per 100,000 individuals, and the reported spontaneous recovery rate varies from 32 to 65%, depending on several prognostic factors (4–7).

The underlying pathophysiology of ISSNHL is believed to involve multiple mechanisms, including viral infections, vascular compromise, immune-mediated damage, and cochlear membrane rupture (5, 7). Due to the unclear etiology, various treatment regimens have been proposed, with systemic corticosteroids being the mainstay therapy. Additional treatments, such as hyperbaric oxygen therapy (HBOT), vasodilators, and antiviral agents, were offered with limited results (8–10).

Over the years, numerous studies have identified prognostic factors that influence recovery in patients with ISSNHL. Some well-documented prognostic factors include the time from onset to treatment initiation, the degree of initial hearing loss, the presence of vertigo, patient age, and the specific audiometric pattern observed at diagnosis. Other factors, such as comorbid conditions (e.g., diabetes mellitus, hypertension) have also been investigated (11–13).

The potential influence of environmental and seasonal factors on the incidence and prognosis of ISSNHL has been a topic of interest in recent literature. Various epidemiological studies conducted across different regions have explored some seasonal patterns in the incidence of ISSNHL (11, 13–26).

Meteorological factors, including temperature fluctuations, humidity, and changes in atmospheric pressure, have been studied for their role in the onset and prognosis of ISSNHL. For example, Zhang et al. (26) found that a high diurnal temperature range and relative humidity were associated with different audiogram configurations, suggesting a possible impact of weather conditions on disease presentation. However, conflicting results from other studies have raised doubts about the significance of weather conditions and seasonal variation in ISSNHL incidence (15, 18).

Despite recent advances in understanding environmental impacts on ISSNHL incidence, research specifically examining seasonality as a prognostic factor for recovery remains limited. In their studies, Narozny et al. (13) and Durmuş et al. (16) observed that patients diagnosed with ISSNHL during the spring season exhibited better hearing recovery than those diagnosed in other seasons. This finding suggests that seasonal factors, such as environmental conditions or seasonal viral activity, may influence recovery outcomes. However, despite identifying a positive correlation, the studies’ conclusions are constrained by relatively small sample sizes (133 and 68 patients) and by the fact that they remain the only studies to report this specific association.

As such, there is a clear need for further investigation into whether the season of onset can serve as a prognostic indicator for hearing recovery outcomes.

This study has two primary objectives: (1) to determine whether ISSNHL incidence demonstrates seasonal variation, and (2) to examine whether the season of onset influences hearing recovery outcomes. Pure-tone average (PTA) improvement serves as the primary outcome measure for recovery, with speech recognition threshold (SRT) and word recognition score (WRS) as secondary outcome measures. This large-scale investigation analyzes 596 patients treated between 2012 and 2023, representing the most extensive cohort study of seasonal effects on ISSNHL incidence and recovery to date.

## Materials and methods

A retrospective cohort study was conducted from 2012 to 2023 at the Department of Otolaryngology and Head and Neck Surgery, Shaare Zedek Medical Center, focusing on hospitalized patients diagnosed with ISSNHL. The study protocol was approved by the institutional review board with a waiver of informed consent.

### Treatment protocol

Treatment followed the AAO-HNS guidelines for patients without contraindications to corticosteroids (1). The protocol consisted of high-dose oral steroids (60 mg prednisone daily) administered for 7 days. Patients showing suboptimal recovery, defined as persistent sensorineural hearing loss of at least 10 dB across two or more frequencies, received salvage therapy with intratympanic steroid injections (one injection daily for seven consecutive days). We included only patients who presented within 14 days of their initial sudden hearing loss, ensuring the cohort represented actual acute cases in which treatment outcomes could be meaningfully assessed.

### Study population and design

Inclusion required a confirmed ISSNHL diagnosis and initial hearing assessment within 2 weeks of symptom onset. Patients were excluded if they had: (1) hearing loss attributable to other causes (such as acoustic trauma, conductive hearing loss, or Ménière’s disease); (2) retrocochlear pathology (vestibular schwannoma or clinical findings suggestive of retrocochlear involvement); (3) age under 18 years; (4) congenital hearing loss; (5) incomplete treatment adherence or inadequate follow-up; or (6) pre-existing severe-to-profound bilateral hearing loss or profound unilateral hearing loss (>90 dB HL) in the contralateral ear that would preclude meaningful assessment of relative hearing improvement.

To assess seasonal variations, patients were categorized into four seasonal groups based on symptom onset: Winter (December through February), spring (March through May), summer (June through August), and autumn (September through November).

Seasonal categorization was based specifically on symptom onset date rather than treatment initiation date to capture the environmental and temporal conditions at disease onset. The 14-day inclusion window (diagnosis within 14 days of symptom onset) was selected based on AAO-HNS clinical practice guidelines recommending treatment initiation within 2 weeks of onset for optimal outcomes (1). This window also serves an important methodological purpose: by limiting the maximum interval between symptom onset and treatment to 14 days, we ensured that both events occurred within the same season or, at most, within adjacent transitional periods, thereby preventing seasonal misclassification.

### Cohort flow and data completeness

During the 12-year study period (2012–2023), 596 patients met inclusion criteria and comprised the final analytical cohort. All patients were managed as inpatients during initial treatment, receiving standardized care according to our institutional protocol. This inpatient approach ensured complete adherence to the treatment regimen, with all patients completing the 7-day oral corticosteroid course under supervised administration. Baseline audiometric assessment was performed within 48 h of admission for all patients. Post-treatment follow-up audiometry was completed for 590 patients (98.8%), with six patients (1.2%) lost to administrative follow-up unrelated to treatment or clinical status. No patients were excluded for incomplete treatment adherence. The structured inpatient protocol with scheduled audiometric assessments minimized missing data and ensured high-quality outcome measurement across the entire cohort.

### Data collection and assessment

Comprehensive patient data included demographics, vascular risk factors (diabetes, hypertension, ischemic heart disease, and smoking status), associated symptoms (tinnitus and vertigo), and the time interval between symptom onset and treatment initiation. The date and season of diagnosis were also recorded for seasonal analysis.

### Audiometric evaluation

All patients underwent at least two comprehensive audiometric assessments (pre- and post-treatment) conducted in soundproof booths by certified audiologists using a calibrated Grason-Stadler (GSI-61/AudioStar Pro) audiometer. These evaluations included pure-tone audiometry, WRS, and SRT.

### Data processing and analysis

Treatment outcomes were evaluated through changes in multiple parameters, including pure tone average (PTA) (calculated as the arithmetic mean of thresholds at 500, 1,000, 2,000, and 4,000 Hz), speech recognition threshold (SRT), word recognition score (WRS), and pure-tone thresholds at frequencies 500, 1,000, 2,000, 4,000, and 6,000 Hz. WRS was assessed using recorded monosyllabic word lists in the patient’s native language (Hebrew, Arabic, or English as appropriate) presented at 40 dB above SRT or at maximum comfortable loudness level when 40 dB above SRT exceeded comfortable listening levels. While standardized phonetically balanced word lists do not currently exist in Hebrew, recorded materials were utilized consistently across all testing sessions to ensure reproducibility, in accordance with international audiological best practices for speech audiometry. We acknowledge that patients presenting with preserved or mildly impaired WRS (≥80%) have limited room for improvement, which may create ceiling effects in outcome analyses. To address this methodological consideration, we performed supplementary analysis restricted to patients with reduced baseline WRS (<80%), where ceiling effects would be minimal, and the potential for improvement would be greatest.

Hearing loss was quantified both absolutely (affected ear alone-AFF) and relatively (comparing affected to contralateral healthy ear).

For absolute SRT measurements, the following equation was applied:


AFFendSRT−AFFbefSRT

For relative measurements:


(AFFendSRT−AFFbefSRT)/(HealthySRT−AFFbefSRT)

Similar calculations were applied for relative PTA and WRS assessments.

### Recovery classification

In accordance with established criteria based on Siegel’s classification and AAO-HNS guidelines (1, 10), hearing recovery was categorized as: (1) Complete recovery, defined as final PTA ≤ 25 dB, representing restoration to normal hearing thresholds; (2) Partial recovery, defined as PTA improvement ≥10 dB from baseline, reflecting clinically meaningful hearing improvement.

### COVID-19 pandemic sensitivity analysis

To assess the robustness of our findings, we conducted a sensitivity analysis comparing pre-pandemic (2012–2019) and pandemic (2020–2023) years. We compared seasonal distribution, baseline characteristics, and treatment outcomes.

### Statistical analysis

Data analysis was performed using SPSS version 25 (IBM^®^ SPSS^®^ Statistics, Chicago, IL, USA). For the primary objective of seasonal incidence pattern, Chi-square goodness-of-fit test was used to compare observed versus expected case distribution across seasons. For the primary objective of seasonal effects on recovery, audiometric outcomes (PTA, SRT, WRS) were compared across seasons using the Kruskal–Wallis *H* test due to the non-normal distribution of improvement scores. Categorical outcome measures (complete recovery, partial recovery) were compared across seasons using Chi-square test or Fisher’s exact test as appropriate. To control for potential confounding variables, we performed: (1) Analysis of Covariance (ANCOVA) to evaluate seasonal effects on PTA improvement while controlling for baseline PTA, age, gender, and time to treatment; (2) Logistic regression to assess the association between season and binary recovery outcomes (complete vs. incomplete recovery) while adjusting for the same covariates. Continuous variables were compared between groups using independent *t*-tests or Mann–Whitney *U* tests as appropriate based on normality assessment. Categorical variables were compared using Chi-square or Fisher’s exact tests. Correlations between continuous variables were assessed using Pearson or Spearman correlation coefficients as appropriate.

*Multiple comparison strategy*: Primary outcomes (PTA, SRT, WRS improvement) were analyzed as independent research questions requiring no multiplicity correction. Post-hoc pairwise comparisons used Dunn’s test with Bonferroni adjustment (*α* = 0.05/6 = 0.008) when omnibus tests were significant. Prognostic factor analysis (7 covariates) applied Bonferroni correction (*α* = 0.05/7 = 0.007). Exploratory analyses were reported unadjusted as hypothesis-generating (Supplementary material 1).

Given the minimal missing data (1.2%), we employed complete case analysis for all statistical analyses.

A *p*-value of ≤0.05 was considered statistically significant for all tests.

### Literature review

A comprehensive literature review was conducted to provide context for our findings and to compare them with existing evidence in the field, using the following key terms: idiopathic sudden sensory neural hearing loss, prognosis of sudden sensory neural hearing loss, incidence of sudden sensory neural hearing loss, and risk factors for sudden sensory neural hearing loss. Four databases were searched: PubMed, EMBASE, Cochrane Central Register of Controlled Trials, and Google Scholar.

## Results

A total of 596 patients diagnosed with ISSNHL were included in the study, distributed across four seasons: 145 (24.3, 95% CI: 20.9–27.8) in spring, 140 (23.5, 95% CI: 20.1–26.9) in summer, 147 (24.7, 95% CI: 21.2–28.1) in autumn, and 164 (27.5, 95% CI: 23.9–31.1) in winter. Chi-square goodness-of-fit test showed no evidence of departure from equal seasonal distribution (*χ*^2^ = 2.188, *p* = 0.534, Cramér’s *V* = 0.061, indicating negligible effect size). There was no evidence of differences in patient demographics, vascular risk factors, associated symptoms (vertigo or tinnitus), or duration from symptom onset to treatment initiation across seasons (Table 1).

**Table 1:** Demographic information, including sex, comorbidities, and associated symptoms, as a function of season of admission.

Season	Spring	Summer	Fall	Winter	*p*-value
Number	145	140	147	164	0.534
M/F	80/65	73/67	78/69	74/90	0.309
Age ± SD (years)	51.48 ± 17.8	52.72 ± 18.3	48.61 ± 17.7	53.44 ± 7.1	0.09
Average days from hearing loss	6.23	6.22	5.97	6.54	0.758
R/L	71/74	62/78	73/74	86/78	0.563
DM %	21	18	25	28	0.69
HTN %	45	37	35	45	0.576
IHD %	12	13	8	13	0.653
Smoking	12	9	12	12	0.903
Tinnitus %	102	97	103	117	0.984
Vertigo %	44	42	50	48	0.814

The *p*-value represents the difference between the four seasons. M/F, male/female; SD, standard deviation; R/L, right/left ears; DM, diabetes mellitus; HTN, hypertension; IHD, ischemic heart disease. *p*-value obtained from Kruskal–Wallis or ANOVA test, as appropriate.

Audiometric parameters at presentation showed no evidence of differences between groups. Baseline SRT ranged from 48.22 dB (winter) to 51.33 dB (fall), and PTA ranged from 43.34 dB (winter) to 45.99 dB (fall). Baseline WRS ranged from 58.48% (fall) to 66.49% (winter). None of these initial audiometric values showed evidence of seasonal differences (*p* > 0.7 for all comparisons) (Table 2).

**Table 2:** Primary audiometry results of the affected ear, and the initial difference from the healthy ear, as a function of season of admission.

Season	Spring	Summer	Fall	Winter	*p*-value
Number	145	140	147	164	0.534
Affected ear first SRT	49.10 ± 31.7	50.39 ± 31.4	51.33 ± 32.9	48.20 ± 29.4	0.827
Affected ear first PTA	45.08 ± 26.6	45.30 ± 25.5	45.99 ± 27.4	43.34 ± 25.1	0.832
Affected ear first WRS (%)	61.1 ± 28.2	60.34 ± 32.9	58.48 ± 27.4	66.49 ± 22.5	0.238
Affected ear first SRT—healthy ear SRT (db)	28.90 ± 34.7	28.79 ± 32.9	32.48 ± 32.9	28.99 ± 30.3	0.721
Affected ear first PTA—healthy ear PTA (db)	26.20 ± 29.7	27.81 ± 26.9	29.34 ± 27.1	26.49 ± 25.7	0.742
Healthy ear WRS—affected ear first WRS (%)	27.94 ± 43.5	26.70 ± 46.0	32.35 ± 43.7	27.41 ± 42.0	0.684

Mean ± SD. The *p*-value (Kruskal–Wallis) represents the difference between the four seasons. SRT, speech recognition threshold; WRS, word recognition score (assessed at 40 dB above SRT or at maximum comfortable loudness level when 40 dB above SRT exceeded comfortable listening levels); dB, decibels; PTA, pure tone average (calculated as the arithmetic mean of thresholds at 500, 1,000, 2,000, and 4,000 Hz).

**Figure 1:**
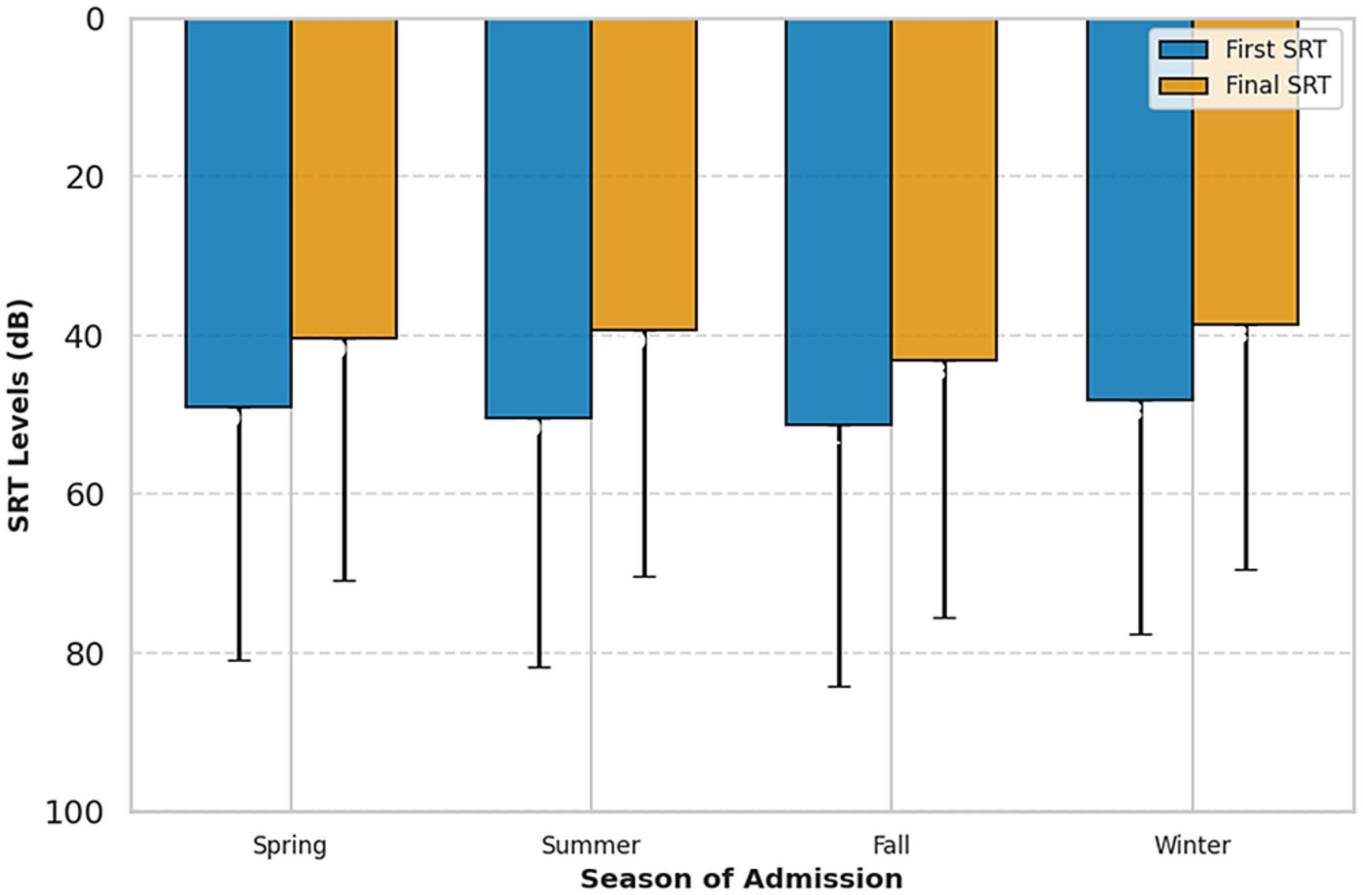
SRT-speech recognition threshold levels of initial and final audiometries, divided into groups based on season of admission, mean + SD.

Post-treatment audiometric parameters demonstrated statistically significant mean improvement across all seasons (*p* < 0.001 compared to baseline for all groups). Mean PTA improvement ranged from 8.62 dB (95% CI: 5.47–11.76) in autumn to 10.48 dB (95% CI: 7.07–13.89) in winter. SRT improvement ranged from 8.16 dB (95% CI: 4.37–11.96) in autumn to 11.04 dB (95% CI: 7.09–14.98) in summer. WRS improvement ranged from 9.40% (95% CI: 4.01–14.79) in winter to 14.15% (95% CI: 8.83–19.47) in autumn. Kruskal–Wallis tests showed no evidence of seasonal differences in improvement magnitude (PTA: *H* = 1.662, *p* = 0.645, *η*^2^ < 0.001; SRT: *H* = 2.110, *p* = 0.550, *η*^2^ < 0.001; WRS: *H* = 4.766, *p* = 0.190, *η*^2^ = 0.003), with overlapping confidence intervals across all seasons and negligible effect sizes (Table 3 and Figure 1). These findings indicate that treatment resulted in significant improvement regardless of season, with no evidence that seasonal timing influenced recovery magnitude. Analysis of recovery classification revealed no significant seasonal variation in either complete recovery rates or partial recovery rates (Table 3).

**Table 3:** Hearing improvement indicators at the end of the treatment according to the season of admissions, mean ± SD.

Season	Spring	Summer	Fall	Winter	*p*-value
Final PTA (db)	36.17 ± 25.5	34.90 ± 26.4	37.37 ± 26.7	32.84 ± 25.9	0.468
PTA improvement: first PTA—final PTA (db)	8.91 ± 16.4	10.39 ± 20.8	8.62 ± 19.4	10.48 ± 22.8	0.784
Final SRT (db)	40.38 ± 30.5	39.36 ± 31.1	43.16 ± 32.4	38.59 ± 30.9	0.604
SRT improvement: first SRT—final SRT (db)	8.72 ± 23.3	11.04 ± 23.8	8.16 ± 23.4	9.60 ± 24.1	0.754
Final WRS (%)	71.53 ± 38.5	73.00 ± 36.6	72.63 ± 38.2	75.73 ± 35.7	0.783
WRS improvement: final WRS—first WRS (%)	10.43 ± 37.7	12.66 ± 36.8	14.15 ± 32.9	9.40 ± 35.1	0.648
Complete recovery, *n* (%)	**62 (42.8)**	**64 (45.7)**	**64 (43.5)**	**87 (53.4)**	**0.216**
Partial recovery, *n* (%)	**57 (39.3)**	**58 (41.4)**	**63 (42.9)**	**72 (44.2)**	**0.847**

The *p*-value (Kruskal–Wallis) represents the difference between the four seasons. SRT, speech recognition threshold; WRS, word recognition score (assessed at 40 dB above SRT or at maximum comfortable loudness level when 40 dB above SRT exceeded comfortable listening levels); dB, decibels; PTA, pure tone average (calculated as the arithmetic mean of thresholds at 500, 1,000, 2,000, and 4,000 Hz). Complete recovery is defined as final PTA ≤ 25 dB; partial recovery is defined as PTA improvement ≥10 dB from baseline.

**Figure 2:**
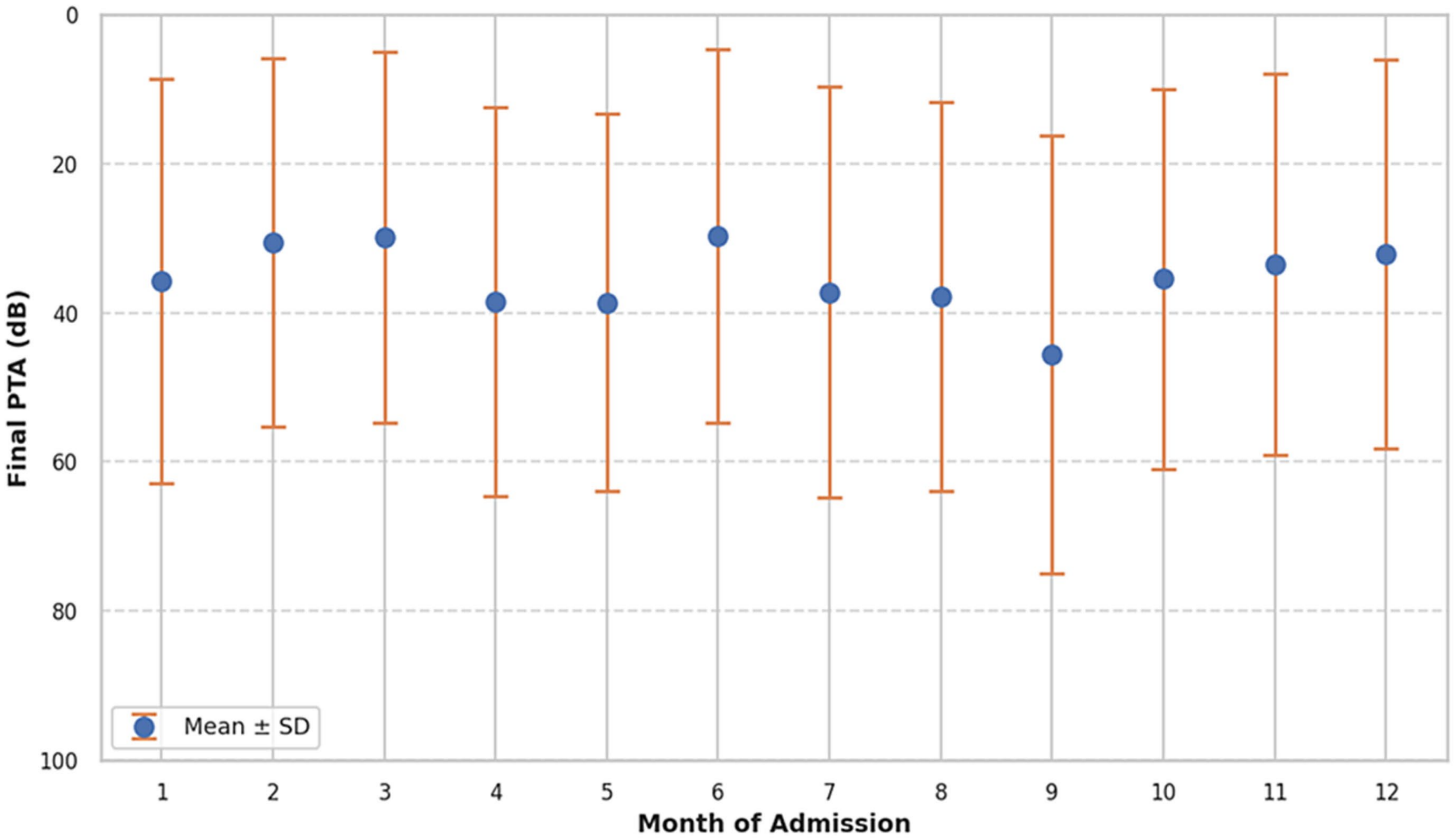
Pure tune average at the end of treatment, divided into groups based on month of admission, mean ± SD.

Monthly analysis of PTA improvement revealed some variation. The highest average PTA improvement was observed in June (15.51 dB), whereas the lowest was seen in September (4.78 dB). Nevertheless, the monthly trends did not demonstrate a consistent seasonal pattern, and inter-month differences were not statistically evaluated (Figure 2).

To address potential confounding by treatment heterogeneity, we examined whether treatment modality (oral corticosteroids alone versus oral plus intratympanic salvage therapy) influenced seasonal effects on recovery. Treatment distribution was balanced across seasons (oral-only: 45–51%; oral + IT: 48–58%; *χ*^2^ = 3.394, *p* = 0.335), indicating no seasonal bias in treatment allocation. Within each treatment modality, seasonal comparisons of PTA improvement revealed no significant differences (oral-only group: *H* = 3.278, *p* = 0.351; oral + IT group: H = 2.129, *p* = 0.546). Mean PTA improvements in the oral-only group ranged from 6.23 dB (summer) to 9.64 dB (winter), while in the oral + IT group they ranged from 8.61 dB (autumn) to 14.31 dB (summer), with no consistent seasonal pattern in either group. These findings demonstrate that the absence of seasonal effects on hearing recovery is consistent across both treatment modalities, indicating that treatment heterogeneity does not confound our primary conclusions.

To address potential ceiling effects in patients with preserved baseline WRS, we performed a sensitivity analysis restricted to the 257 patients (43.1%) with baseline WRS < 80%. In this subgroup with reduced baseline WRS, mean WRS improvement by season was: spring 35.0 ± 39.2% (*n* = 58), summer 34.8 ± 37.2% (*n* = 64), autumn 30.9 ± 40.5% (*n* = 67), and winter 30.6 ± 38.4% (*n* = 68). Kruskal–Wallis analysis revealed no significant seasonal differences in WRS improvement even in this subgroup with greater potential for improvement (*H* = 0.618, *p* = 0.892), consistent with the findings in the full cohort.

COVID-19 Pandemic Sensitivity Analysis comparing pre-pandemic (2012–2019, *n* = 403, 67.6%) and pandemic (2020–2023, *n* = 193, 32.4%) periods revealed no evidence of differences in seasonal distribution (*χ*^2^ = 5.871, *p* = 0.118) or yearly incidence rates (50.4 ± 13.9 vs. 48.2 ± 15.8 patients/year, *p* = 0.798). Baseline disease severity was comparable between periods, with similar PTA (44.1 ± 25.8 vs. 46.6 ± 26.9 dB, *p* = 0.315), and WRS (64.0 ± 41.9 vs. 57.1 ± 44.1%, *p* = 0.113). Time from symptom onset to treatment was significantly shorter during the pandemic period (6.5 ± 4.7 vs. 5.7 ± 4.6 days, *p* = 0.039). PTA improvement (9.2 ± 19.5 vs. 10.4 ± 20.7 dB, *p* = 0.476) was comparable between periods, while WRS improvement was significantly greater in the pandemic period (9.4 ± 34.1 vs. 16.2 ± 38.4%, *p* = 0.003) (Table 4).

**Table 4:** Comparison of pre-pandemic and pandemic periods in idiopathic sudden sensorineural hearing loss patients.

Parameter	Pre-COVID (2012–2019) *n* = 403 (67.6%)	COVID (2020–2023) *n* = 193 (32.4%)	*p*-value
Seasonal distribution			
Spring, *n* (%)	87 (21.6)	58 (30.1)	
Summer, *n* (%)	94 (23.3)	46 (23.8)	
Autumn, *n* (%)	105 (26.1)	42 (21.8)	
Winter, *n* (%)	117 (29.0)	47 (24.4)	
Chi-square test			0.118
Yearly incidence (patients/year)	50.4 ± 13.9	48.2 ± 15.8	0.798
Baseline disease severity			
PTA (dB), mean ± SD	44.1 ± 25.8	46.6 ± 26.9	0.315
SRT (dB), mean ± SD	48.7 ± 31.0	51.9 ± 32.0	0.291
WRS (%), mean ± SD	64.0 ± 41.9	57.1 ± 44.1	0.113
Time to treatment (days) ± SD	6.5 ± 4.7	5.7 ± 4.6	0.039*
Treatment outcomes			
PTA improvement (dB)	9.2 ± 19.5	10.4 ± 20.7	0.476
SRT improvement (dB)	8.6 ± 23.6	11.1 ± 23.8	0.122
WRS improvement (%)	9.4 ± 34.1	16.2 ± 38.4	0.003*

SRT, speech recognition threshold; WRS, word recognition score (assessed at 40 dB above SRT or at maximum comfortable loudness level when 40 dB above SRT exceeded comfortable listening levels); dB, decibels; PTA, pure tone average (calculated as the arithmetic mean of thresholds at 500, 1,000, 2,000, and 4,000 Hz). Statistical comparisons performed using Chi-square test for categorical variables (seasonal distribution) and Mann–Whitney *U* test for continuous variables (all other parameters). Data presented as mean ± standard deviation unless otherwise specified. *Statistically significant (*p* < 0.05).

Finally, prognostic analysis revealed no evidence of impact of sex, diabetes, hypertension, smoking, tinnitus, or vertigo on PTA improvement. The only factor associated with a statistically significant adverse effect on recovery was the presence of ischemic heart disease (IHD), with a PTA improvement of 3.4 dB compared to 10.15 dB in those without IHD (*p* = 0.027). However, following Bonferroni’s adjustment, no evidence of difference remained (Table 5).

**Table 5:** Pure tone average (PTA) improvement with possible risk factors (positive) vs. without possible risk factors (negative).

PTA Improvement with possible risk factors	Positive	Negetive	*p*-value
Male	10.59	8.59	0.221
DM	9.95	9.57	0.869
HTN	9.79	9.57	0.902
IHD	3.4	10.15	**0.027**
Smoking	10.52	9.69	0.79
Tinitus	9.06	10.94	0.294
Vertigo	8.65	10.05	0.429

The *p*-value represents the difference between patients with and without risk factors, as determined by chi-square tests for each risk factor. DM, diabetes mellitus; HTN, hypertension; IHD, ischemic heart disease. Values reaching significance (*p* < 0.05) are displayed in bold.

After controlling for potential confounders, season of onset remained non-significant as a predictor of hearing recovery. ANCOVA evaluating seasonal effects on PTA improvement, while controlling for baseline PTA, age, gender, and time to treatment (*n* = 595), revealed no evidence of a seasonal effect (*F* = 0.710, *p* = 0.546, partial *η*^2^ = 0.004). Adjusted mean PTA improvements ranged from 9.01 dB (Autumn) to 11.75 dB (Winter), with overlapping 95% confidence intervals. Similarly, logistic regression for complete recovery (final PTA ≤ 25 dB) showed no evidence of seasonal differences, with odds ratios versus Winter of: Spring OR = 0.591 (95% CI: 0.341–1.026, *p* = 0.061), Summer OR = 0.744 (0.429–1.288, *p* = 0.291), and Autumn OR = 0.619 (0.357–1.073, *p* = 0.088). Both models confirmed baseline PTA as the strongest predictor (ANCOVA: *F* = 100.717, *p* < 0.001; Logistic: OR = 0.941, p < 0.001), while age, gender, and treatment timing showed no evidence of effects (Supplementary material 2).

A comprehensive literature review identified 14 studies examining seasonal patterns in ISSNHL, encompassing over 60,000 cases across multiple geographical regions (Table 6). Among these studies, 11 investigated seasonal variations in ISSNHL incidence only, while three studies specifically evaluated seasonal effects on recovery outcomes. The studies examining incidence patterns reported mixed findings: Wu et al. (25) and Kim et al. (19) found highest incidence in autumn, Simani et al. (23) and Durmuş et al. (16) reported increased incidence in winter and spring, respectively, while seven studies found no statistically significant seasonal variation in incidence. Regarding prognostic implications, only three studies assessed seasonal effects on hearing recovery outcomes. Narozny et al. (13) (*n* = 133) and Durmuş et al. (16) (*n* = 68) reported better recovery outcomes for spring-onset cases, while Körpinar et al. (20) (*n* = 90) found no significant seasonal effect on prognosis. The remaining studies did not examine seasonal effects on treatment outcomes, focusing solely on incidence patterns.

**Table 6:** Systematic literature review of seasonal patterns in ISSNHL: incidence and prognostic implications.

Reference no.	Author (year)	Country	Cases included	Seasonal distribution (cases per season)	Statistically significant seasonal variation in incidence?	Season implication on prognosis?	Explanation of prognosis implications
(13)	Narozny et al. (2006)	Poland	133	Winter 34 Spring 43 Summer 33 Autumn 23	Higher in spring in one group	Evaluated	Better prognosis and improved recovery if ISSNHL occurred in spring
(20)	Körpinar et al. (2010)	Turkey	90	Winter 15 Spring 27 Summer 22 Autumn 16	No	Evaluated	No significant seasonal effect on prognosis was found
(16)	Durmuş et al. (2018)	Turkey	68	Winter: 16 Spring: 27 Summer: 18 Autumn: 7	Higher incidence in spring	Evaluated	Better recovery in warmer seasons (summer and spring)
(11)	Atay et al. (2016)	Turkey	181	Winter 39 Spring 56 Summer 39 Autumn 47	No	Not evaluated	
(14)	Chang et al. (2005)	Taiwan	146	Winter 26 Spring 51 Summer 28 Autumn 41	No	Not evaluated	
(18)	Jourdy et al. (2010)	USA	97	Winter 34 Spring 43 Summer 33 Autumn 23	No	Not evaluated	
(26)	Zhang et al. (2021)	China	510	Winter 155 Spring 131 Summer 118 Autumn 106	No	Not evaluated	
(15)	Danielides et al. (2002)	Greece	82	Winter 21 Spring 24 Summer 17 Autumn 30	No	Not evaluated	
(25)	Wu et al. (2006)	Taiwan	9,267	Winter: 0.633 Spring: 0.666 Summer: 0.723 Autumn: 0.73. (per 100,000)	Highest ISSNHL incidence in autumn	Not evaluated	
(19)	Kim et al. (2017)	South Korea	45,277 population-based	Winter: 1.36 Spring: 1.28 Summer: 1.65 Autumn: 1.57. (per 100,000)	Highest ISSNHL incidence in autumn	Not evaluated	
(22)	Nakashima et al. (2014)	Japan	4,753 population-based	Winter: 936 Spring: 966 Summer: 1094 Autumn: 1103	No	Not evaluated	
(24)	Tal et al. (2023)	Israel	740	Winter: 200 Spring:170 Summer: 178 Autumn: 192	No	Not evaluated	
(23)	Simani et al. (2022)	Israel	320	Winter: 60 Spring: 90 Summer: 79 Autumn: 91	Higher incidence in winter	Not evaluated	
(17)	Gerçeker et al. (2011)	Turkey	164	Winter: 38 Spring: 52 Summer: 27 Autumn: 47	No	Not evaluated	
	Our study	Israel	596	Winter 164 Spring 145 Summer 140 Autumn 147	No	Evaluated	No significant seasonal effect on prognosis found

## Discussion

This large-scale cohort study provides compelling evidence that the seasonal timing of ISSNHL onset does not significantly influence incidence or treatment outcomes. Our analysis of 596 patients—the largest single-center cohort examining seasonal effects on ISSNHL recovery—demonstrates that while audiometric improvement occurs across all seasons, the season of onset is not an independent predictor of recovery rates or the magnitude of hearing improvement after controlling for relevant clinical variables.

Regarding seasonal incidence patterns, our cohort shows no significant seasonal variation in ISSNHL occurrence, with a nearly uniform distribution across the four seasons (spring: 24.3%, summer: 23.5%, autumn: 24.7%, winter: 27.5%; *p* = 0.534). This finding is particularly noteworthy given our substantial sample size and extended observation period spanning multiple complete seasonal cycles, which provides greater statistical power than many previous investigations. Our results contrast with several reports in the literature suggesting seasonal peaks in ISSNHL incidence. Wu et al. (25) and Kim et al. (19) reported increased ISSNHL incidence in autumn in their respective populations, whereas Simani et al. (23) observed a higher incidence in winter in Israel. Additionally, Narozny et al. (13) and Durmuş et al. (16) both reported a higher incidence in spring. However, seven other studies examining this question found no statistically significant seasonal variation in incidence (11, 14, 15, 17, 18, 22, 24), consistent with our findings. The apparent inconsistencies across studies may reflect geographical differences in climate patterns, varying sample sizes with insufficient statistical power, or regional differences in viral epidemiology and environmental exposures. Our null finding regarding seasonal incidence, derived from one of the largest single-center cohorts examining this question, suggests that if seasonal environmental factors do influence ISSNHL onset, their effect is either negligible or inconsistent across different populations and climatic zones.

Our findings directly challenge the positive seasonal effect reported by Narozny et al. (13) and Durmuş et al. (16). The only prior studies to suggest that spring onset correlated with superior recovery outcomes. Based on relatively small cohorts (*n* = 133 and 68, respectively), their conclusions likely lacked sufficient statistical power to control for potential confounders and year-specific variations. In contrast, our substantially larger dataset, spanning multiple complete seasonal cycles, provides more robust statistical evidence against a meaningful seasonal prognostic effect.

The results of our study align with other investigations that specifically evaluated seasonal influence on ISSNHL recovery. Despite using different treatment protocols, Körpinar et al. (20) similarly reported no significant seasonal impact on auditory recovery following ISSNHL. This consistency across diverse patient populations and treatment approaches reinforces the conclusion that seasonal factors do not meaningfully modify recovery potential.

It is important to distinguish seasonal patterns in disease incidence from seasonal effects on recovery outcomes. The fact that our study found no seasonal influence on either incidence or prognosis suggests that distinct pathophysiological mechanisms may govern disease onset versus recovery. While environmental factors such as viral infections or vascular compromise might theoretically trigger ISSNHL, the biological processes governing cochlear recovery appear to operate independently from these potential seasonal triggers. The relationship between meteorological factors and ISSNHL has been previously investigated, with Zhang et al. (26) identifying correlations between temperature fluctuations, humidity, and specific audiometric configurations. However, our data suggest that while environmental conditions may potentially play a role in disease presentation in some populations, they do not appear to modulate either the incidence in our region or the biological recovery mechanisms. Subsequent recovery is likely to depend more on intrinsic cellular repair mechanisms, inflammatory resolution pathways, and timely medical intervention than on environmental conditions at the time of disease onset.

An important consideration in evaluating the generalizability of our findings relates to the climatic characteristics of our study region. While it is located in a Mediterranean climate, Jerusalem and its surrounding areas experience substantial seasonal temperature variations comparable to those in many temperate climate zones worldwide. Winter temperatures in Jerusalem regularly drop to 5–8 °C at night with daytime averages around 10–12 °C, while summer temperatures exceed 20 °C at night with daytime peaks often reaching 30–35 °C. These pronounced seasonal differences, with temperature ranges exceeding 25 °C between winter and summer, provide sufficient climatic variation to detect seasonal effects if they were truly present. The fact that we observed no significant seasonal influence on either ISSNHL incidence or recovery outcomes despite these marked temperature fluctuations strengthens the validity of our findings across diverse climatic settings. Furthermore, previous studies reporting seasonal effects, including those by Narozny et al. (13) and Durmuş et al. (16), were conducted in regions with similar or even less climatic variability, yet reached different conclusions with substantially smaller sample sizes (*n* = 133 and *n* = 68, respectively).

An important consideration is the potential impact of the COVID-19 pandemic on our findings, as our study period encompasses three pandemic and post pandemic years (2020–2023). Recent studies have reported associations between SARS-CoV-2 infection and ISSNHL (27, 28), as well as possible vaccine-related auditory complications (29), raising questions about whether pandemic-era ISSNHL cases differ from pre-pandemic presentations. Our sensitivity analysis comparing the pre-pandemic (2012–2019) and pandemic (2020–2023) periods revealed consistent seasonal patterns, comparable annual incidence rates, and similar baseline severity and treatment outcomes across eras (Table 4). Notably, patients during the pandemic presented significantly earlier for treatment and demonstrated greater WRS improvement, possibly reflecting the established relationship between early treatment initiation and functional outcomes (1, 30). The consistency of both seasonal patterns and disease incidence across periods supports the robustness of our primary conclusions, though the pandemic’s role as a potential confounder in seasonal ISSNHL studies warrants consideration in future research.

This study presents a comprehensive literature review table (Table 6) that examines explicitly seasonal effects on ISSNHL recovery outcomes. While several large-scale studies from East Asia, such as those by Wu et al. (25), Kim et al. (19), and Nakashima et al. (22), have evaluated seasonal variations in ISSNHL incidence using national healthcare databases, these investigations have significant limitations. Despite their impressive sample sizes, these registry-based studies lack detailed audiometric outcome data, preventing analysis of actual hearing recovery metrics. Our study addresses this critical gap by providing both large-scale epidemiological data and comprehensive audiometric outcomes, enabling a more nuanced understanding of seasonal influences on recovery parameters rather than merely incidence patterns.

Interestingly, other clinical variables previously suggested as prognostic indicators, including diabetes, hypertension, vertigo, and tinnitus (11, 12), did not significantly impact treatment response in our cohort after controlling for age and treatment delay.

Certain limitations warrant consideration. As a single-center retrospective study, our design is inherently subject to selection bias and documentation inconsistencies. Additionally, while our analysis controlled for major clinical variables, we did not have access to detailed virological or immunological data that might have provided further insights into potential seasonal mechanisms.

Our findings have important clinical implications. Clinicians should be reassured that the timing of ISSNHL onset within the calendar year does not necessitate modification of prognostic expectations or treatment approaches. Instead, established factors such as prompt intervention, degree of initial hearing loss, and audiometric configuration remain the key determinants of recovery potential (5, 11, 12).

Future research directions should include prospective multicenter studies incorporating detailed meteorological, virological, and immunological variables to elucidate further the complex environmental and host factors that may contribute to both ISSNHL onset and recovery.

## Conclusion

This large-scale cohort study of 596 patients provides compelling evidence that the seasonal onset of ISSNHL does not significantly influence incidence or hearing recovery outcomes despite minor variations observed across different months and seasons. Clinicians should be reassured that the timing of ISSNHL onset within the calendar year does not necessitate modification of prognostic expectations or treatment approaches, allowing focus to remain on well-established factors such as prompt intervention and initial hearing loss severity.

## Data Availability

The raw data supporting the conclusions of this article will be made available by the authors, without undue reservation.
