# Supplementary material for: Seasonal effects on incidence and outcomes in idiopathic sudden sensorineural hearing loss
Source: Front Neurol. 2026 Mar 23;17:1753066. doi: 10.3389/fneur.2026.1753066 (PMC13050685; doi:10.3389/fneur.2026.1753066)
Supplement: Supplementary file 1 [file Table_1.docx]

**Supplementary 1. Multiple Comparison Strategy and Corrections Applied**

| Analysis Family | Specific Tests | Number of Tests | Correction Method | Adjusted α | Classification |
| --- | --- | --- | --- | --- | --- |
| Primary Objective 1: Seasonal Incidence | Chi-square goodness-of-fit for seasonal distribution | 1 | None | 0.050 | Primary |
| Primary Objective 2: Seasonal Effects on Recovery | Kruskal-Wallis tests: • PTA improvement • SRT improvement • WRS improvement | 3 | None | 0.050 each | Primary |
| Secondary Outcomes: Recovery Classification | Chi-square tests: • Complete recovery rate • Partial recovery rate | 2 | None | 0.050 each | Secondary |
| Post-hoc Pairwise Comparisons | Dunn's test following significant Kruskal-Wallis (6 pairwise comparisons per outcome) | 6 per outcome | Bonferroni | 0.050/6 = 0.008 | Primary/Secondary |
| Prognostic Factor Analysis | Univariate comparisons: • Sex • Diabetes • Hypertension • IHD • Smoking • Tinnitus • Vertigo | 7 | Bonferroni | 0.050/7 = 0.007 | Secondary |
| Adjusted Models | ANCOVA and Logistic Regression (seasonal effect after controlling for covariates) | 2 | None | 0.050 each | Secondary |
| Frequency-Specific Outcomes | Seasonal comparisons at: 500, 1000, 2000, 4000, 6000 Hz | 5 | None | 0.050 | Exploratory |
| Subgroup Analyses | Seasonal effects by: • Baseline severity • Treatment timing • Treatment modality | Multiple | None | 0.050 | Exploratory |
| COVID-19 Sensitivity | Pre-pandemic vs pandemic period comparisons | Multiple | None | 0.050 | Exploratory |

**Rationale for Correction Strategy:**

- Primary outcomes (seasonal incidence and three audiometric recovery measures) were pre-specified and considered independent research questions, requiring no correction for multiplicity.
- Post-hoc pairwise comparisons were only performed when omnibus tests (Kruskal-Wallis) reached significance, with Bonferroni correction applied to control family-wise error rate within each outcome.
- Prognostic factor analysis examined 7 pre-specified covariates as potential predictors of recovery, requiring Bonferroni correction to maintain overall α=0.05.
- Adjusted models (ANCOVA and logistic regression) tested the same primary hypothesis (seasonal effect) using different statistical approaches to control for confounders, considered as confirmatory sensitivity analyses without additional correction.
- Exploratory analyses were explicitly designated as hypothesis-generating and reported without correction, with interpretation appropriately cautious regarding multiple testing.
- This strategy balances protection against Type I error inflation with preservation of statistical power for pre-specified primary comparisons, following current best practices in clinical research (Perneger, 1998; Rothman, 1990).
