# Supplementary material for: Seasonal effects on incidence and outcomes in idiopathic sudden sensorineural hearing loss
Source: Front Neurol. 2026 Mar 23;17:1753066. doi: 10.3389/fneur.2026.1753066 (PMC13050685; doi:10.3389/fneur.2026.1753066)
Supplement: Supplementary file 2 [file Table_2.docx]

**Supplementary 2: Adjusted Analyses Results**

ANCOVA and Logistic Regression Models for Seasonal Effects

**1. ANCOVA: Seasonal Effect on PTA Improvement (Adjusted)**

| Variable | F-statistic | P-value | Partial η² |
| --- | --- | --- | --- |
| Season (primary variable) | 0.710 | 0.546 | 0.004 |
| Baseline PTA | 100.717 | <0.001 | 0.146 |
| Age | 0.449 | 0.503 | 0.001 |
| Gender | 0.943 | 0.332 | 0.002 |
| Time to treatment | 2.275 | 0.132 | 0.004 |

- Model fit: R² = 0.153, Adjusted R² = 0.142, F = 15.099, p < 0.001
- Sample size: n = 595

**Adjusted Means by Season (LSMeans):**

- Spring: 9.51 dB (95% CI: 6.22-12.80)
- Summer: 10.94 dB (95% CI: 7.56-14.32)
- Autumn: 9.01 dB (95% CI: 5.70-12.31)
- Winter: 11.75 dB (95% CI: 8.47-15.02)

**2. Logistic Regression: Seasonal Effect on Complete Recovery (Adjusted)**

| Season (vs Winter) | Odds Ratio | 95% CI | P-value |
| --- | --- | --- | --- |
| Spring | 0.591 | 0.341-1.026 | 0.061 |
| Summer | 0.744 | 0.429-1.288 | 0.291 |
| Autumn | 0.619 | 0.357-1.073 | 0.088 |
| Winter | 1.000 (reference) | — | — |

**Other Covariates in Model:**

- Baseline PTA: OR = 0.941 (95% CI: 0.918-0.965), p = <0.001
- Age: OR = 1.000 (95% CI: 0.989-1.011), p = 0.941
- Gender (Female vs Male): OR = 0.910 (95% CI: 0.620-1.336), p = 0.640
- Time to treatment: OR = 0.980 (95% CI: 0.938-1.023), p = 0.346
- Model fit statistics:
- Nagelkerke R² = 0.418
- Cox & Snell R² = 0.313
- AIC = 615.41, BIC = 650.54
- Classification accuracy = 76.2%
- Sample size: n = 596
- Complete recovery cases: 277 (46.5%)

Complete recovery defined as final PTA ≤25 dB. All models control for baseline PTA, age, gender, and time from symptom onset to treatment initiation. ANCOVA uses Type II sums of squares. Logistic regression uses maximum likelihood estimation with Winter as the reference season. Partial η² represents the proportion of variance in the outcome uniquely explained by each predictor.
